# Membranous E-Cadherin Expression in Different Subtypes of Pituitary Neuroendocrine Tumors and Its Association with Invasiveness

**DOI:** 10.3390/ijms27062672

**Published:** 2026-03-14

**Authors:** Anna Krzentowska, Beata Biesaga, Anna Merklinger-Gruchała, Filip Gołkowski

**Affiliations:** 1 Department of Endocrinology and Internal Medicine, Andrzej Frycz Modrzewski Krakow University Medical College, 30-705 Kraków, Poland; fgolkowski@uafm.edu.pl; 2 Department of Medical Biology, Andrzej Frycz Modrzewski Krakow University Medical College, 30-705 Kraków, Poland; bbiesaga@uafm.edu.pl; 3 Department of Bioinformatics and Public Health, Andrzej Frycz Modrzewski Krakow University Medical College, 30-705 Kraków, Poland; amerklinger-gruchala@uafm.edu.pl

**Keywords:** membrane E-cadherin, pituitary adenoma, PitNETs, invasiveness, transcriptions factors

## Abstract

Pituitary neuroendocrine tumors (PitNETs) are usually benign intracranial neoplasms that may exhibit invasion of the cavernous sinus, complicating surgery and increasing the risk of recurrence. This study aimed to investigate membranous E-cadherin (mE-cad) expression across PitNET subtypes and transcription factor (TF) lineages, including Pit-1 (pituitary-specific positive transcription factor 1), SF-1 (Steroidogenic Factor 1), and TPIT (T-box pituitary transcription factor), and its association with tumor invasiveness in sixty-nine patients. mE-cad expression was evaluated as the percentage of positive cells (0%, 1–10%, >10%) and by immunoreactive score (IRS). Staining intensity was scored as: 0, no staining; 1, weak; 2, moderate; 3, strong. The proportion of positive cells was scored as: 0, none; 1, <10%; 2, 10–50%; 3, 51–80%; 4, >80%. Mean mE-cad expression was 5.2% in gonadotroph, 3.2% in corticotroph, 0.5% in lactotroph, and 17.5% in plurihormonal PitNETs. By TF lineage, the mean expression was 5.3% for Pit-1, 3.2% for TPIT, and 5.1% for SF-1. Low mE-cad expression (IRS 1–2) was associated with higher odds of cavernous sinus invasion compared with IRS 3–6 (adjusted OR = 6.0, 95% CI 1.08–33.4, *p* = 0.04), independent of tumor volume (adjusted OR = 4.0, 95% CI 1.50–10.7, *p* = 0.01). After restricting the analysis to the gonadotroph PitNET group, tumors with an IRS of 1–2 showed significantly higher invasiveness compared with those with an IRS of 3–6 (*p* = 0.012). These findings suggest that mE-cad may serve as a biomarker of PitNET invasiveness, with expression varying according to TF lineage and tumor subtype.

## 1. Introduction

PitNETs are among the most common intracranial tumors, accounting for approximately 15% of all intracranial neoplasms [1]. These tumors are classified into several categories, including hormonally active and inactive, as well as invasive and non-invasive. According to the 2022 World Health Organization (WHO) classification of pituitary tumors, they are further categorized based on the transcription factor lineage from which they originate [2]. Hormonally active tumors produce clinical symptoms resulting from excess hormone secretion and are therefore often detected at an earlier stage. In contrast, hormonally inactive tumors, which do not cause endocrine symptoms, tend to reach larger sizes and lead to manifestations related to mass effect and invasion of adjacent structures [3].

Numerous studies have investigated the impact of various biomarkers on the biological behavior of pituitary tumors. One such biomarker is E-cad, encoded by the *CDH1* gene located on chromosome 16q22.1. E-cad is a member of the cadherin family of transmembrane adhesion proteins that mediate calcium-dependent cell–cell interactions [4]. Their primary function is to facilitate adhesion between cells of the same type. The intracellular domain of cadherins interacts with proteins of the catenin family. The E-cad–catenin complex regulates cell adhesion and motility and acts as a suppressor of cellular invasion. Loss of cadherin-mediated cell–cell adhesion may alter the biological behavior of PitNETs [5]. Molecular alterations associated with epithelial–mesenchymal transition may also contribute to PitNET recurrence [6,7].

Previous studies have reported decreased expressions of E-cad, β-catenin, and H-cadherins in PitNETs, whereas increased expressions of N-cadherin and catenins have been observed [6,8]. Numerous investigations have evaluated the role of E-cad in the invasiveness and recurrence of pituitary tumors, with inconsistent findings. Several studies have demonstrated reduced E-cad expression in invasive tumors [6,9]; however, other reports have found no significant association between E-cad expression and tumor invasiveness [10] or recurrence [10,11]. E-cad expression has been analyzed across different PitNET subtypes, including lactotroph [12,13], corticotroph [14,15], somatotroph [16,17,18], and hormonally inactive tumors [19], particularly gonadotroph tumors [19,20,21], as well as mixed lactotroph/somatotroph tumors [22]. Some studies have suggested that reduced E-cad expression may be associated with a more aggressive clinical course of these tumors [8,13].

The subcellular localization of E-cad is critical for its evaluation. Membranous expression, assessed by immunohistochemistry (IHC) and quantified using the immunoreactive score (IRS), reflects its adhesive function. Reduced membranous expression may indicate loss of cell–cell adhesion and increased migratory potential. Nuclear expression, evaluated by IHC targeting the intracellular domain, may reflect the translocation of E-cad from the membrane to the nucleus and may influence cellular behavior. Assessment of total E-cad localization (membranous, cytoplasmic, and nuclear), performed at the mRNA level using quantitative PCR (qPCR) or by immunofluorescence, enables analysis of CDH1 gene expression in the context of epithelial–mesenchymal transition and cellular phenotype. Several studies have highlighted the translocation of E-cad from the cell membrane to the nucleus and its potential impact on tumor biology [19].

The tumor microenvironment has also been recognized as a key factor influencing tumor behavior, particularly through the activity of chemokines. Silva et al. demonstrated that CCL2 (C–C motif chemokine ligand 2) mRNA expression was negatively correlated with *CDH1* expression and total membranous E-cad staining [23].

In light of the above findings, we analyzed mE-cad expression in a cohort of patients who underwent surgery for pituitary tumors.

Primary objective: To assess differences in mE-cad expression across PitNET subtypes and according to transcription factor lineage, including Pit-1, SF-1, and TPIT.

Secondary objective: To evaluate the association between mE-cad expression and tumor invasiveness in PitNETs.

## 2. Results

### 2.1. Characteristics of All Patients Who Underwent Surgery for PitNETs (n = 69)

Among the 69 patients enrolled, 43 were men (62.3%) and 26 were women (37.7%). The mean age ± SD of the patients was 57.8 (13.7). The median mE-cad expression [%] was 1.25% (Q1–Q3: 0–11 %), min–max: 0.0–36.3%. The distribution was right-skewed, with most values clustered at low levels. Similarly, the median of IRS score for mE-cad was 1.0 (Q1–Q3: 0.0–4.0), min–max: 0.0–6.0, and also showed right-skewed distribution. The detailed results concerning epidemiological and clinical data are presented in Table 1.

### 2.2. Analysis of the Association Between mE-cad Expression and Tumor Type, and Between mE-cad Expression and TFs Status

The mean mE-cad expression was evaluated according to PitNET subtype and TF status. The lowest mE-cad expression was observed in corticotroph (mean 3.2%, *n* = 8), lactotroph (mean 0.5%, *n* = 3), gonadotroph (mean 5.2%, *n* = 40) and thyrotroph (single observed value 0.0%, *n* = 1). In null cell adenomas (*n* = 2), mE-cad expression ranged from 0% to 5%. Higher expression levels were observed in somatotroph (single observed value 15%, *n* = 1) and plurihormonal tumors (mean 17.5%, *n* = 3).

**Figure 1:**
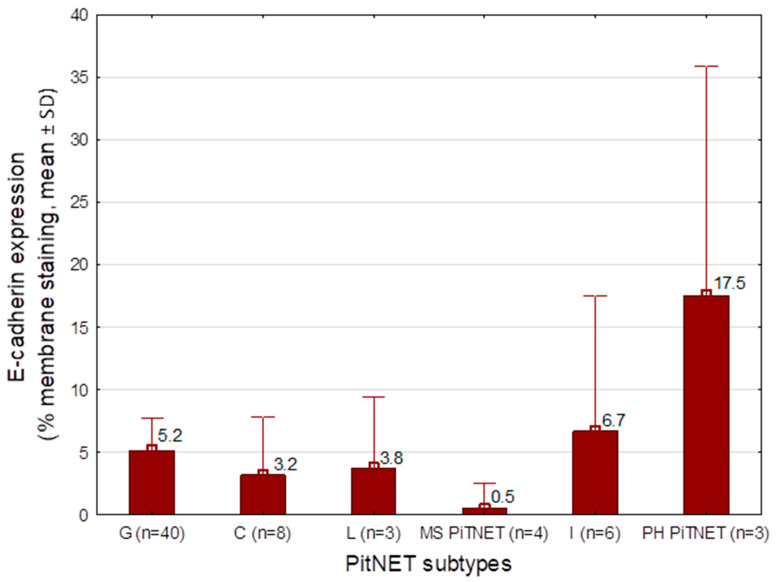
mE-cad expression (% membranous staining, mean ± SD) across different PitNET subtypes: G, gonadotroph; G/L, gonadotroph/lactotroph; C, corticotroph; L, lactotroph; MS PitNET, multiple synchronous PitNET; I, immature PIT-lineage tumor; PH PitNET, plurihormonal PITNET. The mean value for each tumor subtype is shown, with whiskers indicating the standard deviation (SD).

**Figure 2:**
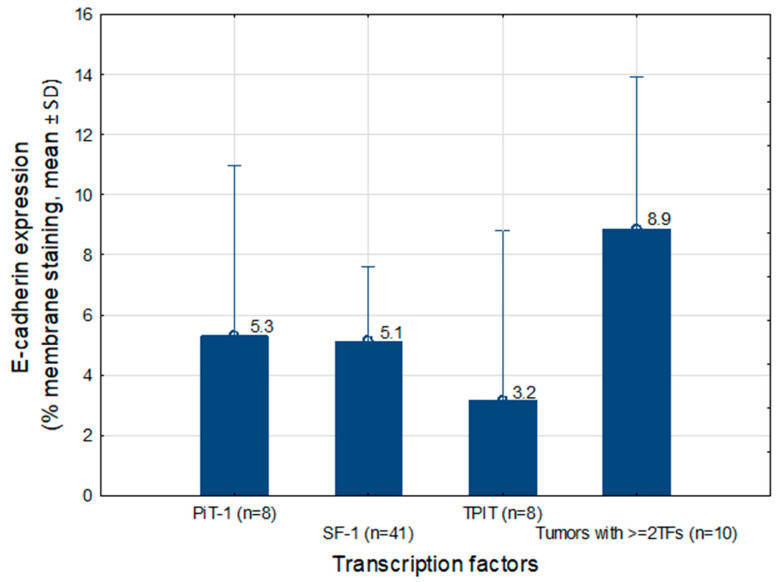
mE-cad expression (% membranous staining, mean ± SD) across transcription factor-defined tumor groups: PIT-1, SF-1, TPIT, and tumors expressing ≥2TFs. The mean value for each transcription factor group is shown, with whiskers indicating the standard deviation (SD).

Analysis of mE-cad expression according to TF status showed the highest levels in tumors expressing ≥2 transcription factors (mean 8.9%, *n* = 10), whereas the lowest expression was observed in tumors of the TPIT lineage (mean 3.2%, *n* = 8). In null cell adenomas (*n* = 2), mE-cad expression values were 0% and 5%. Only mean values for PitNET subtypes or TF groups with *n* ≥ 3 are presented in Figure 1 and Figure 2.

### 2.3. Analysis of mE-cad Expression Broken Down into Absent (0%), Weak (1–10%), and Moderate (>10%), and According to IRS Categories (0, 1–2, 3–6)

Subsequently, mE-cad expression was categorized into three groups, absent (0%), weak (1–10%), and moderate (>10%), based on the right-skewed distribution of the data. Tumors with weak or absent mE-cad expression were found to have a significantly larger tumor volume compared with tumors showing higher levels of expression (*p* = 0.03). Other epidemiological and clinical characteristics did not vary according to mE-cad expression levels. Likewise, no significant differences in clinical features (age, sex, hormonal activity) or tumor characteristics (volume, maximum size, transcription factor profile, and PitNET subtype) were observed between mE-cad IRS categories. However, a trend toward larger tumor volume was observed in the lower IRS categories (*p* = 0.06). The results are presented in Table 2.

### 2.4. Analysis of the Association Between mE-cad Expression and Cavernous Sinus Invasion According to the Knosp Scale

The relationship between mE-cad expression and tumor invasiveness was evaluated. Tumors were classified as non-invasive if they belonged to Knosp grades 0–2, and as invasive if they belonged to grades 3–4. The expression of mE-cad was expressed according to three categories: 0, 1–10%, and >10%. In our study group, the IRS was found in the range from 0 to 6. The relationship between IRS (0, 1–2, 3–6) and tumor invasiveness in the entire study group was analyzed. The distribution of mE-cad expression according to cavernous sinus invasion is presented in Table 3.

In the analysis of the relationship between IRS values and PitNETs invasiveness (according to the Knosp scale), significant differences were observed between groups: lower IRS ranges, i.e., IRS 1–2, predominated in invasive tumors, whilst higher IRS were more frequent in non-invasive tumors, and the IRS 0 was similarly distributed across groups (Holm–Bonferroni correction *p* = 0.04). A similar trend was observed for mE-cad expression: invasive tumors more frequently exhibited low mE-cad expression, i.e., the 1–10% category (Holm–Bonferroni correction *p* = 0.064).

To determine whether the association between IRS and tumor invasiveness was independent of tumor size, multivariable logistic regression modeling was performed (Table 4).

Logistic regression analyses showed that tumors with low mE-cad expression (IRS 1–2) had significantly higher odds of cavernous sinus invasion compared with tumors with high expression (IRS ≥ 3–6) in both crude (OR = 6.24, 95% CI: 1.44–27.06, *p* = 0.014) and adjusted models controlling for tumor volume (OR = 6.01, 95% CI: 1.08–33.42, *p* = 0.041). Tumors with complete loss of expression (IRS = 0) showed a similar trend but did not reach significance (adjusted OR = 3.07, 95% CI: 0.69–13.65, *p* = 0.14). Tumor volume was independently associated with invasion (adjusted OR = 4.00, 95% CI: 1.50–10.70, *p* = 0.006). The multivariable model demonstrated good fit (Hosmer–Lemeshow *p* = 0.48; AUC = 0.80) and explained a moderate proportion of variance in cavernous sinus invasion (Cox–Snell R^2^ = 0.26; Nagelkerke R^2^ = 0.35).

### 2.5. Analysis of mE-cad Expression in Gonadotroph PitNETs

The largest subgroup in our cohort consisted of gonadotroph tumors. The impact of mE-cad on invasiveness was assessed within this homogeneous group. After adjustment for multiple testing using the Holm–Bonferroni method, both the IRS categories and the percentage of mE-cad-positive cells were significantly associated with tumor invasiveness according to the Knosp scale (IRS: adjusted *p* = 0.012; mE-cad %: adjusted *p* = 0.014), supporting the robustness of these findings in this tumor subtype. Invasive tumors predominated among cases with low IRS scores (1–2), whereas complete loss of mE-cad expression (IRS = 0) was evenly distributed between non-invasive and invasive tumors. High IRS scores (3–6) were more frequent among non-invasive tumors. Similar associations were observed for the percentage of mE-cad-positive cells, with low expression (1–10%) being more common in invasive tumors and higher expression (>10%) more frequent in non-invasive tumors. The results are presented in Table 5.

In this subgroup of gonadotroph tumors, the results of logistic regression analyses before and after adjustment to tumor volume were consistent with those observed in the entire cohort. Tumors with IRS 1–2 showed higher odds of invasion compared with tumors with high expression (IRS ≥ 3–6; crude *p* = 0.007, adjusted *p* = 0.013). However, due to the limited sample size and low number of events, effect estimates were imprecise and should be interpreted with caution.

## 3. Discussion

mE-cad is a key adhesion protein that maintains intercellular cohesion. Immunohistochemical analyses have shown that E-cad expression may be heterogeneous. Physiologically, E-cad localizes to the cell membrane, whereas aberrant cytoplasmic expression reflects disturbances in protein maturation and function. Nuclear localization, although rare, has been associated with more aggressive tumor behavior.

Previous studies have reported variable patterns of E-cad expression in pituitary adenomas, ranging from typical membranous localization that supports cell–cell adhesion to focal or attenuated expression, and even complete loss, which may correlate with a more aggressive tumor phenotype. In the present study, complete loss of E-cad expression was observed in 34 of 69 cases (49.2%), which is comparable to the results reported by Stefanidis et al. (34/94) and Qian et al. (21/69, 30.0%) [8,10]. Within our cohort, complete loss of mE-cad expression was observed in 48.8% of gonadotroph tumors and 60% of corticotroph tumors.

Our analysis also revealed an association between mE-cad expression and tumor volume: tumors with absent or low expression (<10%) were significantly larger than those with higher expression levels. Similar associations between reduced E-cad expression and tumor size have been described in the literature [4,18], although some studies did not demonstrate such a relationship [10].

Analysis of the relationship between mE-cad expression and tumor invasiveness in our cohort showed that invasive tumors more frequently exhibited a low (1–2) immunoreactive score (IRS). A similar trend was observed for the percentage of positive cells: low mE-cad expression (1–10%) predominated in invasive tumors. Interestingly, the highest odds of cavernous sinus invasion were observed in tumors with low but detectable mE-cad expression (IRS 1–2) rather than in tumors with complete loss of expression. Although the estimate for IRS = 0 showed a similar trend, it did not reach statistical significance, likely due to the limited sample size and wide confidence intervals. This pattern may reflect biological heterogeneity among tumors lacking mE-cad expression or differences in the mechanisms regulating cell adhesion and invasiveness. A sensitivity analysis restricted to gonadotroph tumors confirmed the same direction of association between low mE-cad expression and cavernous sinus invasion as observed in the overall cohort.

These findings are consistent with the biological role of mE-cad, in which reduced cell–cell adhesion facilitates tumor invasion. Several studies have investigated the association between E-cad expression and pituitary tumor invasiveness [9,10]. Some reports confirmed that invasive tumors exhibit lower E-cad expression compared with non-invasive tumors [9], whereas other studies did not observe such a relationship [10]. It should be noted that variations in the assessment of membranous and/or nuclear expression across studies may have influenced these results. Table 6 summarizes studies evaluating the association between E-cad expression and tumor invasiveness.

Several studies have evaluated the membranous and nuclear expression of E-cad [19,22,25,26,27,29]. Loss of membranous E-cad, accompanied by nuclear localization, may contribute to a more invasive or aggressive pituitary tumor phenotype. This mechanism is related to epithelial–mesenchymal transition (EMT), during which reduced cell–cell adhesion facilitates cellular migration. Previous studies have reported that E-cad is often lost from the cell membrane while being detected in the nucleus, suggesting that extracellular domain cleavage and nuclear translocation of E-cad is a common phenomenon that may drive invasion in pituitary adenomas [26]. Similarly, other studies have indicated that nuclear accumulation of E-cad, together with loss of membranous expression, correlates with tumor invasiveness [25,29]. In the study by Berardinelli et al. [27], increased protein expression and nuclear translocation were observed in NF-PitNETs. Both E-cad and KLHL14 (Kelch-like family member 14) shifted from cytoplasmic localization in low-invasive NF-PitNETs to nuclear localization in highly invasive NF-PitNETs. This study suggests a novel intracellular mechanism regulating the tumor’s propensity for local invasion. In the study by Gil et al. [30], the expression of genes associated with EMT was assessed at the mRNA level using quantitative PCR (qPCR). EMT marker expression was more pronounced in NF-PitNETs compared with somatotroph PitNETs, suggesting a stronger EMT phenotype in these non-functioning tumors.

Analyzing mE-cad expression according to PitNET subtype in our study, we found that the lowest levels of mE-cad were observed in corticotroph tumors (mean 3.2%), thyrotroph tumors (0.0%), lactotroph tumors (mean 0.5%), and gonadotroph tumors (mean 5.2%). The literature has also assessed E-cad expression across different pituitary tumor subtypes.

Several studies have focused on lactotroph PitNETs [12,13,22,31,32], including tumors with aggressive behavior [13,31,32], highlighting that reduced expression of the E-cad/catenin complex may influence tumor behavior [13]. Reduced E-cad expression has been confirmed in invasive lactotroph tumors compared with non-invasive ones [12], as well as altered E-cad localization in invasive tumors [22].

Regarding corticotroph tumors in our study, five out of eight tumors of this type showed no mE-cad expression, with a mean expression of 3.2%. Analyses of E-cad expression in these tumors reported in the literature show varying results [14,15,19,33]. In the study by Kiseljak-Vassiliades et al., molecular analysis of corticotroph subtypes demonstrated that E-cad immunohistochemistry was positive in 80%, diminished in 17%, and absent in 20%, with no correlation to corticotroph PitNET subtype, size, or prognosis [14]. Corticotroph tumor progression has been confirmed to be associated with reduced expression of the epithelial marker E-cad [15]. Comparisons of E-cad expression in subgroups of non-functioning pituitary neuroendocrine tumors revealed that corticotroph NF-PitNETs exhibited a higher IRS for both the extracellular and intracellular domains of E-cad than gonadotroph NF-PitNETs [19].

Somatotroph PitNETs: In our study, there was a single case of a somatotroph tumor, with an mE-cad expression of 15%. Previous studies on somatotroph PitNETs have analyzed E-cad expression [28,34,35,36,37,38] and its association with responses to somatostatin receptor ligands (SRLs) [28,34,35,36]. E-cad has been reported as the best molecular predictor of responses to SRLs [28]. Densely granulated tumors with positive SSTR2 and E-cad expression have been suggested to be associated with favorable SRL responses [36]. Based on several studies, E-cad may serve as a practical marker for predicting SRL responsiveness [17,28,35].

Gonadotroph PitNETs lack reliable prognostic markers predicting their clinical course. In our study, the mean expression of mE-cad in gonadotroph tumors was 5.2%. A statistically significant difference was observed between invasive and non-invasive tumors in this subgroup. Notably, in our study, invasive tumors exhibited absent or low (1–10%) mE-cad expression in 70% and 100% of cases, respectively. This may explain the tendency of these tumors to reach large sizes, often several centimeters, which prevents complete resection and frequently necessitates additional surgical interventions. Studies in the literature have also focused on the expression of this protein in this tumor type [19,20,21]. E-cad expression has been examined among non-functioning tumors, suggesting that high N-cadherin levels and reduced membranous E-cad expression are not associated with more aggressive behavior in these NF-PitNET subgroups [19]. In the study by Kolnes et al., NF-PitNETs with high FSH expression exhibited reduced membranous E-cad staining and increased nuclear E-cad staining, whereas LH expression was not associated with E-cad [20]. Similarly, Øystese et al. demonstrated almost no immunoreactivity for the extracellular domain of E-cad in silent gonadotroph tumors [21].

Analyzing mE-cad expression according to transcription factor (TF) status in our study, the lowest values were observed in null cell adenomas (2.5%), slightly higher in TPIT-lineage tumors, and the highest in tumors expressing ≥2 TFs. In the study by Øystese et al., the distribution of E-cad across different cell lineages was examined, showing that corticotroph NF-PitNETs (lineage TPIT) exhibited higher levels of E-cad (both extracellular and intracellular domains) compared with gonadotroph PitNETs (lineage SF-1). In contrast, null cell adenomas showed low mE-cad expression and variable intracellular expression, which is consistent with our findings [19]. The variation in mE-cad expression depending on TF status suggests that E-cad levels reflect the degree of differentiation and the complexity of the tumor’s transcriptional program, rather than a straightforward aggressive potential.

In summary, the literature reports studies supporting a relationship between reduced or mislocalized (nuclear) E-cad expression and tumor invasiveness [6,8,18,27,29]. At the same time, some studies did not observe a significant correlation in selected cohorts [25,26], which may be attributable to methodological heterogeneity (e.g., IHC, cut-off values, and tumor subtype). Differences in IHC evaluation (membranous versus nuclear localization), the antibodies and scoring systems employed, and the types of pituitary adenomas studied (GH-, PRL-, or NF-PitNETs) may account for these inconsistencies and complicate direct quantitative and statistical comparisons.

The results of our study indicate that mE-cad demonstrates only a moderate capacity to differentiate tumors according to their degree of invasiveness. Although this marker does not simultaneously provide high sensitivity and specificity, it may serve as a complementary tool for evaluating the invasiveness of gonadotroph tumors and provide additional information when combined with other molecular and clinical parameters.

Finally, the limitations of our study should be acknowledged. The heterogeneity of the study cohort and the small number of certain tumor subtypes, sometimes represented by only a single observation, constitute a limitation and require cautious interpretation of the results, particularly with regard to subgroup analyses. Nevertheless, we believe that our study contributes to understanding the role of E-cad in PitNETs, which may ultimately facilitate improved treatment for patients affected by this rare disease.

## 4. Material and Methods

### 4.1. Characteristics of Study Group

Retrospective immunohistochemical and radiological analysis of PitNETs was performed. The study included a group of 69 patients who underwent surgery at St. Raphael’s Hospital in Krakow, Poland, between 2022 and 2024, who were referred for surgery for a tumor within the sella turcica, and in whom a pituitary tumor was subsequently confirmed via histopathology (HP). Each patient gave informed consent for the collection of tumor tissue for the study. The patient data were anonymized. The study was approved by the Bioethics Committee of Andrzej Frycz Modrzewski Krakow University (permission No KBKA/31/O/2024 issued on 6 June 2024).

### 4.2. Classification of Pituitary Neuroendocrine Tumors (PitNETs)

The PitNETs included in this study were classified according to the 2022 World Health Organization classification of endocrine and neuroendocrine tumors (5th edition, beta website version). The classification was based on immunohistochemical (IHC) staining for pituitary hormones (growth hormone (GH), prolactin (PRL), adrenocorticotropic hormone (ACTH), follicle-stimulating hormone/luteinizing hormone (FSH/LH), and thyroid-stimulating hormone (TSH)) and the expression of lineage-specific TFs: Pit-1, SF-1 and TPIT. Tumors were categorized into the following lineages and subtypes: PIT1 lineage: somatotroph, lactotroph, thyrotroph, and pluri-hormonal PIT1-positive tumors; TPIT lineage: corticotroph tumors; SF1 lineage: gonadotroph tumors. Null cell tumors are those lacking both hormone and transcription factor expression. In a few cases, the tumors showed ≥ 2TFs. Only tumors with available and interpretable IHC results for both hormonal and transcription factor markers were included in the subtype analysis.

### 4.3. Magnetic Resonance Imaging of the PitNETs

Each patient was subjected to a pituitary-targeted magnetic resonance imaging (MRI) scan before surgery. Radiological assessment was based on pituitary MRI with contrast. The thickness of the layers used was 2–3 mm. All standard MRI sequences necessary to diagnose pituitary adenoma were used. Measurements were performed using contrast-enhanced T1-weighted images. Based on the MRI image, the tumor was measured in 3 dimensions, i.e., AP, ML, and CC (cor x sag x cc), and the tumor volume was calculated. The tumor invasion into the cavernous sinuses was assessed using the Knosp scale: Grade 0: The tumor does not cross the medial line of the internal carotid artery. Grade 1: The tumor is confined medial to the intercavernous line, crossing the vertical meridian of the carotid siphon in the cross-section. Grade 2: Tumors extend past the intercavernous line but stay within the line tangent to the supracavernous and intracavernous carotid arteries. Grade 3: Tumors spread lateral to the lateral tangential line. Grade 4: Tumors totally encase the intracavernous carotid artery.

Diagnostic criteria of invasive PitNETs: In our study, tumors of Knosp grades 0 and 1 were classified as non-invasive, while grade 2, 3 and 4 tumors were classified as invasive. A total of 69 consecutive patients underwent transsphenoidal excision of the pituitary tumor via the transnasal approach. All of the operations were performed by the same neurosurgeon in St. Raphael’s Hospital in Krakow.

### 4.4. Immunohistochemical Assessment

#### 4.4.1. Immunohistochemical Assessment of the Hormones and Transcription Factors

Immunohistochemical assessment of TFs and hormones was performed according to the methods described in our previous study on pituitary tumors, published in 2025 in the International Journal of Molecular Sciences [39].

#### 4.4.2. Immunohistochemistry of mE-Cad

Formalin-fixed, paraffin-embedded (FFPE) tumor blocks were cut into 4 µm sections using a rotary microtome. The sections were transferred onto Superfrost Plus™ microscope slides (Gerhard Menzel, Glasbearbeitungswerk GmbH & Co. KG, Braunschweig, Germany) and left to dry overnight at 37 °C. Subsequently, the slides were heated at 60 °C for 1 h prior to immunostaining. Paraffin removal was achieved by immersing the slides in three successive xylene baths (5 min each), followed by stepwise rehydration in decreasing concentrations of ethanol (2 × 100% and 1 × 95%). Antigen retrieval was carried out by heat-induced epitope unmasking in citrate buffer (Target Retrieval Solution, pH 6.1; DAKO Cytomation, Glostrup, Denmark) at 96 °C for 50 min. After retrieval, the slides were allowed to cool slowly to room temperature while remaining in the buffer and were then rinsed with distilled water. Endogenous peroxidase activity was blocked by incubating the sections in a solution of 3% hydrogen peroxide in methanol for 30 min. To minimize non-specific antibody binding, the slides were incubated with eBioscience™ IHC/ICC High Protein Blocking Buffer (Thermo Fisher Scientific) for 5 min at room temperature in a humidified chamber. Primary antibodies (E-cad Monoclonal Antibody, no cat. 4A2C7, TermoFisher Scientific–Invitrogen, Waltham, MA, USA) diluted according to the manufacturers’ recommendations were applied to the sections and incubated overnight at 4 °C in a moisture-controlled chamber. Immunoreactions were visualized using the Bright Vision detection system (Immunologic, Duiven, The Netherlands) in combination with 0.01% 3,3′-diaminobenzidine tetrahydrochloride (Vector Laboratories, Burlingame, CA, USA). The sections were counterstained with Mayer’s hematoxylin for 1 min, rinsed under running tap water for 10 min, dehydrated through graded alcohol solutions, cleared in xylene, and permanently mounted using a synthetic mounting medium. Each staining series included both a known E-cad-positive tissue (such as tonsil or placenta) as a positive control and a negative control in which the primary antibody was omitted.

**Figure 3:**
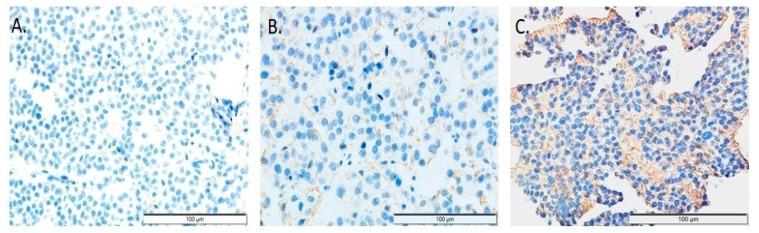
Representative microphotographs of immunohistochemical staining for E-cad in PitNETs. (**A**) Lack of membranous E-cad expression. (**B**) Weak, focal membranous immunoreactivity. (**C**) Moderate and diffuse membranous E-cad expression. Original magnification: 400×.

E-cad expression was evaluated according to previously published criteria [8,17]. The applied antibody demonstrated predominantly membranous staining, with occasional cytoplasmic immunoreactivity; however, in the present cohort, no cytoplasmic staining was observed. Positive immunohistochemical reactivity was detected exclusively as membranous staining of tumor cells; therefore, only membranous E-cad expression was considered positive and included in the analysis (Figure 3). During microscopic evaluation, staining intensity was also assessed using a four-point scale: 0, no staining; 1, weak staining; 2, moderate staining; and 3, strong staining.

mE-cad results were reported as the percentage of tumor cells exhibiting membranous expression and were additionally quantified using the immunoreactive score (IRS). The IRS was calculated by multiplying the proportion of positively stained tumor cells (0 = none, 1 = <10%, 2 = 10–50%, 3 = 51–80%, 4 = >80%) by the staining intensity score, resulting in a total score ranging from 0 to 12. Based on classifications commonly applied in the literature [19], E-cad expression was categorized as absent (IRS = 0), weak (IRS = 1–2), moderate (IRS = 3–8), or strong (IRS = 9–12).

### 4.5. Statistical Analysis

Continuous variables were reported as mean with standard deviation (SD) and median with interquartile range (Q1–Q3), and compared using the Mann–Whitney U test or Kruskal–Wallis test. The normality of data distributions was assessed using the Shapiro–Wilk test. Categorical variables were presented as counts and percentages, and compared using the Pearson Chi-square test. ROC analysis was performed to indicate the cut-off point for the expression of E-cad, assuming an invasive tumor on the Knosp scale (3–4) as the outcome. The cut-off points were determined by Youden’s method. Logistic regression analyses were performed to evaluate the association between mE-cad expression (IRS categories) and cavernous sinus invasion according to the Knosp classification. Both crude (univariable) and adjusted (multivariable) models were calculated. In the multivariable model, adjustment was made for tumor volume (z-score). All analyses were conducted using available data (complete-case analysis), with no imputation applied for missing values. Because two alternative classifications of mE-cad expression (percentage of positive cells and IRS categories) were evaluated, *p*-values were adjusted for multiple testing using the Holm–Bonferroni method. In all other analyses, a *p*-value of less than 0.05 was considered statistically significant. Calculations were performed using Statistica version 14.1.0.4 (TIBCO Software Inc., Palo Alto, CA, USA).

## 5. Conclusions

Our findings indicate that reduced mE-cad expression correlates with PitNETs invasiveness and varies according to tumor subtype and TF status. However, the results of our study should be interpreted with caution due to the heterogeneity of the cohort and the small sample size of specific tumor types.

## Figures and Tables

**Table 1:** Demographic, radiological and immunohistochemical characteristics of the study group (*n* = 69).

Characteristic	N (%)
Age (years) Mean (SD)/median (Q1–Q3)	57.1 (13.7)/60.0 (47.0–68.0)
Gender, *n* (%) F/M	26 (37.7%)/43 (62.3%)
Tumor volume (cm^3^) Mean (SD)/median (Q1–Q3)	8.5 (9.2)/4.6 (2.9–10.2)
Max size of tumor (mm) Mean (SD)/median (Q1–Q3)	28.5 (10.3)/25.0 (21.3–33.0)
Transcription factors, *n* (%) Pit-1 SF-1 TPIT Null cell adenoma PitNETs with ≥2TFs	8 (11.6%) 41 (59.4%) 8 (11.6%) 2 (2.9%) 10 (14.5%)
Hormonal activity, *n* (%) Non-active Active	54 (78.3%) 15 (21.7%)
Type of PitNET, *n* (%) Gonadotroph Gonadotroph/lactotroph Corticotroph Lactotroph Somatotroph Thyrotroph Null cell adenoma Multiple synchronous tumor Immature PIT-1 lineage tumor Mature PIT-1 lineage tumor	40 (58.0%) 1 (1.4%) 8 (11.6%) 3 (4.3%) 1 (1.4%) 1 (1.4%) 2 (2.9%) 4 (5.8%) 6 (8.7%) 3 (4.3%)
mE-cad expression, *n* (%) Mean (SD)/median (Q1–Q3) Minimum/maximum	5.38 (7.88)/1.25 (0.0–11.0) 0.0/36.3
Knosp scale (cavernous sinus invasion) Invasive (Knosp 3–4) Non-invasive (Knosp 0–2) Missing	34 (49.3%) 33 (47.8%) 2 (2.9%)

**Table 2:** Assessment of mE-cad expression levels and IRS according to demographic characteristics, tumor size and volume, tumor type, and TF status.

**Characteristic**	mE-cad Expression (*n* = 69)			*p-* Value	mE-cad IRS (*n* = 69)			*p-* Value
	0%	1–10%	>10%		IRS (0)	IRS (1–2)	IRS (3–6)	
	(*n* = 34)	(*n* = 17)	(*n* = 18)		(*n* = 32)	(*n* = 19)	(*n* = 18)	
Age (years) ^a^				0.27				0.30
Mean (SD)	55.4 (12.8)	56.5 (18.1)	61.9 (10.4)		54.8 (13.0)	57.3 (17.3)	61.1 (9.9)	
Me (Q1–Q3)	57.5 (44.0–67.0)	63.0 (41.0–71.0)	62.0 (55.0–70.0)		55.5 (43.5–67.0)	64.0 (41.0–71.0)	62.0 (55.0–69.0)	
Gender, *n* (%)				0.33				0.59
F/M	11 (42.3)/23 (52.3)	9 (34.6)/8 (18.2)	6 (23.1)/12 (27.9)		11 (42.3)/21 (48.8)	9 (34.6)/10 (23.3)	6 (23.1)/12 (27.9)	
Tumor volume (cm^3^) ^a^				0.03				0.06
Mean (SD)	7.7 (9.1)	13.0 (10.4)	5.8 (6.4)		8.0 (9.2)	11.8 (10.4)	5.8 (6.6)	
Me (Q1–Q3)	4.8 (3.2–9.0)	9.5 (4.0–20.5)	3.2 (1.6–5.2)		5.0 (3.5–9.0)	8.8 (3.3–20.0)	3.2 (1.5–8.5)	
Max. size of tumor (mm) ^a^				0.28				0.27
Mean (SD)	28.6 (9.9)	31.4 (10.4)	26.3 (10.8)		29.0 (10.1)	30.4 (10.2)	25.8 (10.9)	
Me (Q1–Q3)	25.0 (22.0–33.0)	31.0 (23.0–39.5)	23.0 (17.0–35.0)		25.5 (22.0–33.0)	29.5 (23.0–36.0)	23.0 (17.0–33.0)	
Hormonal activity of PitNET, *n* (%) ^b^				0.30				0.24
Non-active	27 (49.1)	15(27.3)	12 (22.2)		25 (46.3)	17 (31.5)	12 (22.2)	
Active	7 (46.7)	2 (13.3)	6 (40.0)		7 (46.7)	2 (13.3)	6 (40.0)	
Transcription factors, *n* (%) ^b^				0.67				0.60
PiT-1	5 (62.5)	1 (12.5)	2 (25.0)		5 (62.5)	1 (12.5)	2 (25.0)	
SF-1	20 (47.6)	11 (26.2)	10 (24.4)		18 (43.9)	13 (31.7)	10 (24.4)	
TPIT	5 (62.5)	2 (25.0)	1 (12.5)		5 (62.5)	2 (25.0)	1 (12.5)	
Null cell adenoma	1 (50.0)	1 (50.0)	0 (0.0)		1 (50.0)	1 (50.0)	0 (0.0)	
PitNETs with ≥2 TFs	3 (30.0)	2 (20.0)	5 (50.0)		3 (30.0)	2 (20.0)	5 (50.0)	
Type of PitNET, *n* (%) ^b^				0.11				0.11
Gonadotroph	20 (48.8)	10 (24.4)	10 (25.0)		18 (45.0)	12 (30.0)	10 (25.0)	
Gonadotroph/lactotroph	0 (0.0)	0 (0.0)	1 (100.0)		0 (0.0)	0 (0.0)	1 (100.0)	
Corticotroph	5 (62.5)	2 (25.0)	1 (12.5)		5 (62.5)	2 (25.0)	1 (12.5)	
Lactotroph	2 (66.7)	1 (33.3)	0 (0.0)		2 (66.7)	1 (33.3)	0 (0.0)	
Somatotroph	0 (0.0)	0 (0.0)	1 (100.0)		0 (0.0)	0 (0.0)	1 (100.0)	
Thyrotroph	1 (100.0)	0 (0.0)	0 (0.0)		1 (100.0)	0 (0.0)	0 (0.0)	
Null cell adenoma	1 (50.0)	1 (50.0)	0 (0.0)		1 (50.0)	1 (50.0)	0 (0.0)	
Multiple synchronous tumor	1 (25.0)	3 (75.0)	0 (0.0)		1 (25.0)	3 (75.0)	0 (0.0)	
Immature PIT-1 lineage tumor	4 (66.7)	0 (0.0)	2 (33.3)		4 (66.7)	0 (0.0)	2 (33.3)	
Mature Pit-1 lineage tumor	0 (0.0)	0 (0.0)	3 (100.0)		0 (0.0)	0 (0.0)	3 (100.0)	

^a^ Kruskal–Wallis test, ^b^ Pearson Chi-square test.

**Table 3:** Analysis of the relationship between the levels of mE-cad expression (%) and IRS and cavernous sinus invasion according to the Knosp scale.

Parameter	Non-Invasive (Knosp 0–2)	Invasive (Knosp 3–4)	*p*-Value ᵡ	*p* -Value (Adjusted) ^£^
mE-cad (0%)	17 (50.0%)	17 (50.0%)	0.032	0.064
mE-cad (1–10%)	4 (25.0%)	12 (75.0%)		
mE-cad (>10%)	12 (70.6%)	5 (29.4%)		
IRS (0)	16 (50.0%)	16 (50.0%)	0.040	0.040
IRS (1–2)	5 (26.2%)	13 (68.4%)		
IRS (3–6)	12 (66.7%)	5 (27.8%)		

ᵡ Pearson Chi-square test; ^£^ after Holm–Bonferroni correction for multiple comparisons; all analyses were performed excluding missing data.

**Table 4:** Univariate (crude) and multivariate (adjusted) logistic regression models assessing predictors of tumor invasiveness according to the Knosp scale. Odds ratios (ORs) and 95% confidence intervals (CIs) are presented.

Predictors	Crude		Adjusted	
	OR (95% CI)	*p-*Value	OR (95% CI)	*p-*Value
IRS 0 vs. ≥3–6	2.40 (0.69–8.40)	0.171	3.07 (0.69–13.65)	0.14
IRS 1–2 vs. ≥3–6	6.24 (1.44–27.06)	0.014	6.01 (1.08–33.42)	0.041
Tumor volume (z-score)	4.22 (1.63–10.95)	0.003	4.00 (1.50–10.70)	0.006

Adjusted model fit statistics: Hosmer–Lemeshow χ^2^ = 7.6, *p* = 0.48; AUC = 0.80 (SE = 0.05); Cox–Snell R^2^ = 0.26; Nagelkerke R^2^ = 0.35.

**Table 5:** Analysis of the relationship between the levels of mE-cad expression (%) and IRS and cavernous sinus invasion according to the Knosp scale in gonadotroph PitNETs (*n* = 40).

Parameter	Non-Invasive (Knosp 0–2) *n* (%)	Invasive (Knosp 3–4) *n* (%)	*p*-Value ᵡ	*p*-Value (Adjusted) ^£^
mE-cad (0%)	10 (50.0%)	10 (50.0%)	0.007	0.014
mE-cad (1–10%)	1 (10.0%)	9 (90.0%)		
mE-cad (>10%)	8 (80.0%)	2 (20.0%)		
IRS (0)	9 (50.0%)	9 (50.0%)	0.012	0.012
IRS (1–2)	2 (16.7%)	10 (83.3%)		
IRS (3–6)	8 (80.0%)	2 (20.0%)		

ᵡ Pearson Chi-square test; ^£^ after Holm–Bonferroni correction for multiple comparisons; all analyses were performed excluding missing data.

**Table 6:** A summary of the results of studies on E-cad expression in the literature.

Author	No of Tumors	Type PitNETs	Location E-Cad	Expression Rating/Scoring of IHC	Effect of E-Cad Expression on Invasiveness
Kawamoto et al. (1997) [24]	30	Different	Membrane	Four-grade scale of positive staining cells: 0, negative; 1+, immunopositive cells observed in <30% of all adenoma cells; 2+, 30–70% of all adenoma cells; 3+, >70% of all adenoma cells.	No significant differences between IPT and NIPT.
Qian et al. (2002) [12]	39	Lactotroph	Membrane	The percentage of positive cells was scored as 0 (none), 1+ (1–20%), 2+ (21–50%), 3+ (51–80%), and 4+ (>80%). Staining intensity was scored as weak (0), moderate (1), or strong (2). The sum of these scores yielded a total immunoreactive score (IRS) ranging from 0 to 6, with tumors scoring 1–3 classified as exhibiting significantly reduced expression.	Significantly lower in IPT than in NIPT.
Qian et al. (2007) [8]	69	Different	Membrane	The percentage of positive cells was scored as 0 (no staining), 1+ (1–20%), 2+ (21–50%), 3+ (51–80%), and 4+ (>80%). Staining intensity was scored as weak (0), moderate (1), or strong (2). The scores were added to obtain a total score ranging from 0 to 6, with tumors scoring 1–3 classified as exhibiting significantly reduced expression.	Significantly lower in IPT (II, III, IV of Knosp scale) than in NIPT (I).
Fougner et al. (2010) [18]	61	Somatotroph	Membrane	The percentage of positively stained cells	Correlation of lower expression with invasiveness.
Zhou et al. (2013) [6]	91	Different	Membrane	IRS	Correlation of lower expression with invasiveness.
Chauvet et al. (2016) [22]	50	Somatotroph/lactotroph	Membrane	IRS	Lower expression in IPT.
Mendes et al. (2017) [25]	35	Somatotroph	Membrane	IRS	No significant correlation with invasiveness.
Ongaratti et al. (2019) [26]	53	NFPitNET	Gene and protein expression	IRS	No significant correlation with invasiveness.
Stefanidis et al. (2022) [10]	94	FPitNET and NFPitNET	Membrane	IRS	No significant correlation with invasiveness.
Øystese K.A.B. et al. (2022) [19]	135	NFPitNET, corticotroph, null -cell	Membrane and nuclear	IRS	No significant correlation with invasiveness.
Berardinelli et al. (2024) [27]	30	NFPitNET	Membrane and nuclear	IRS	Correlation of nuclear expression with higher invasiveness.
Kiseljak-Vassiliades et al. (2024) [14]	77	Corticotroph	Membrane + cytoplasmic	IRS	No correlation between E-Cad expression and the invasiveness.
Puig-Domingo et al. (2019) [28]	55	Somatotroph	Membrane	IRS	Correlation of low expression E-cad with weaker response to SSA.

Legend: IHC, immunohistochemistry; IRS, immunoreactive score; IPT, invasive pituitary tumor; NIPT, non-invasive pituitary tumor, NFPitNET, non-functioning pituitary adenomas, FPitNET, functioning pituitary adenomas; SSA, somatostatin analogs.

## Data Availability

The original contributions presented in this study are included in the article. Further inquiries can be directed to the corresponding author.
